# Effect of acupuncture on rehabilitation treatment of children with different degrees of autism spectrum disorder: a randomized controlled trial

**DOI:** 10.3389/fpsyt.2026.1849124

**Published:** 2026-06-10

**Authors:** Qinliang Zhang, Mo Li, Zhenwei Zhou, Liyan Tian, Lianju Liu, Jun Zhang

**Affiliations:** 1 Department of Children’s Rehabilitation, Linyi People’s Hospital, Linyi, China; 2 Epilepsy Sleep Center, Linyi People’s Hospital, Linyi, China

**Keywords:** autism spectrum disorder, CARS, children, psychoeducational profile third edition, scalp acupuncture

## Abstract

**Introduction:**

We investigated the effect of scalp and tongue acupuncture on the rehabilitation of children with autism spectrum disorder (ASD) and the difference in the effect between mild-to-moderate and severe children of ASD.

**Methods:**

Children in all groups received routine rehabilitation training, including educational and therapeutic measures, while the acupuncture treatment groups also received scalp and tongue acupuncture.

**Results:**

Regardless of whether acupuncture treatment is used, rehabilitation training measures have a significant effect on improving the ability of children with ASD. These results are reflected in both PEP-3 and CARS scores. Moreover, compared to usual treatment, acupuncture led to a greater reduction in CARS scores and significant PEP-3-based improvements in cognition, language, motor skills, social behavior, and self-care. In the children of the mild-to-moderate treatment group, acupuncture positively affected cognition verbal/preverbal, expression language, receptive language, visual-motor imitation, social reciprocity, and adaptive behavior. Additionally, among children with severe ASD, acupuncture improved cognition, language, and problem behavior compared to controls. No side effects were observed in the children of the acupuncture group.

**Conclusion:**

Scalp and tongue acupuncture significantly improved cognition, language, motor skills, social behavior, and self-care in children with ASD, with treatment effects varying by severity.

**Clinical Trial Registration:**

https://www.chictr.org.cn/showproj.html?proj=39184, identifier ChiCTR1900023247.

## 1 Introduction

Autism spectrum disorder (ASD) is a neurodevelopmental disorder that starts in early childhood and lasts a lifetime. The core symptoms of ASD include social dysfunction, stereotypical behavior, and a narrow range of interests (1). The prevalence of ASD has increased dramatically over the past few decades, from 1 in 5–000 in 1975 to 1 in 31 in 2022 (2). Although the specific etiology remains unknown, the factors affecting ASD include prenatal factors, neuroanatomical abnormalities, environmental factors, and genetic factors (3, 4). As a specific cause is lacking, unified etiological treatment measures are unavailable (5). However, “early detection, early treatment” is an accepted treatment principle. The main treatment measures for ASD are based on behavioral and educational approaches. Additionally, drug treatment and complementary and alternative medicine (CAM) treatments might be administered. About 40% of children with ASD are administered CAM treatment, of which acupuncture is the most common type (6). Many studies have reported that acupuncture can improve the symptoms of children with ASD, and it has been used for treating children with ASD in many countries as a complementary and alternative treatment (7, 8). The summary of the study found that acupuncture is effective and safe in the treatment of ASD (9–11), and the combined treatment of tongue acupuncture is beneficial to improve the symptoms of ASD (12). A good disease rating level is the key to determining the effectiveness of acupuncture treatment. The Children Autism Rating Scale (CARS) and the Psychoeducational Profile-Third Edition (PEP-3) are commonly used for assessing ASD (9). As a diagnostic scale for evaluating ASD, the CARS score is used extensively to diagnose childhood autism and categorize it into mild-to-moderate and severe types (13, 14). PEP-3 is a scale for evaluating the effect of treatment on autism and determining the scores for different aspects in children with autism, for example, communication, movement, behavioral characteristics. It is based on structured teaching, which mainly includes the evaluation by doctors and the role of caregivers (15). The PEP-3 assessment is frequently used for assessing the development of children with autism but is rarely used for assessing the effectiveness of acupuncture in the treatment of autism (15). Acupuncture has a specific therapeutic effect on children with ASD, but the differences between mild-to-moderate and severe children of ASD in the effects are yet to be elucidated. In this study, children with ASD were first categorized based on their CARS scores, and then, they were administered comprehensive scalp and tongue acupuncture treatments. Finally, their PEP-3 scores were evaluated to determine the effects of the treatment. The specific effects of scalp acupuncture and tongue acupuncture on children with varying severity of ASD were evaluated. We hypothesized that acupuncture would exert a beneficial promotional effect on improving the clinical symptoms of children with ASD.

## 2 Methods

### 2.1 Study design

The study is a 21 weeks RCT that conducted in September 2019 to December 2020 from ASD children who underwent rehabilitationtraining in Linyi People’s Hospital, Shandong, China.

### 2.2 Participants

All children with ASD who underwent rehabilitation training at the Department of Children’s Rehabilitation, Linyi People’s Hospital, Shandong, China, from September 2019 to December 2020, and satisfied the diagnostic criteria of the American Diagnostic and Statistical Manual of Mental Disorders (DSM-V) were eligible to participate in this study. The CARS was used to assess the severity of the condition (13). All ASD diagnoses were made by the attending physician with extensive clinical experience according to the DSM-V diagnostic criteria in combination with the CARS assessment scale.

The inclusion criteria were as follows: (1) Children aged 2–6 years. (2) Prior to this treatment, there was no rehabilitation training or acupuncture therapy. Because the past treatment may affect the treatment effect in the later period, which has an impact on the comparability of the study. (3) All participating children had to meet DSM-V diagnostic criteria for ASD. (4) All children who participated in the treatment had CARS scores above 30 in order to be more consistent with the diagnosis of ASD. (5) Guardians of the children voluntarily participated in the trial and provided written informed consent.

The exclusion criteria were as follows: (1) Complicated with family history of psychosis, schizophrenia or severe intellectual disability; (2) Combined with major organ dysfunction; (3) Combined with coagulation dysfunction; (4) Combined with hearing or physical disability; (5) Unable to cooperate with acupuncture treatment.

### 2.3 Ethical considerations

This study was approved by the Chinese Clinical Trial Registry (registration number: ChiCTR1900023247) and the Ethics Committee of Linyi People’s Hospital (Medical Review No. YX200227). The guardians of all participants were fully informed about the trial and were asked to sign the consent form to let their children participate in this study.

### 2.4 Sample size

We determined the power of the sample size through calculations using the GPower 3.1 software (16). The results of the power analysis indicated that 108 (acupuncture group = 54, control group = 54) participants were needed for a power of 0.85 (α = 0.05, effect size d = 0.52), reference literature for result analysis (16). Accounting for the potential loss of participants, we estimated that 120 participants would be sufficient to accurately determine the effect size.

### 2.5 Randomization and blinding

The stratified block randomization technique was used to randomly place the eligible patients into the acupuncture group or the control treatment group, randomized grouping as per the article (17). To ensure a balance between the groups, we stratified the CARS scores (mild-to-moderate and severe ASD), with a block size of 4. A random number table was constructed using Microsoft Excel. The random numbers were sealed in opaque envelopes with continuous numbers written outside. The envelopes were arranged in the order of screening. The researcher (not the authors) who screened the participants opened the envelopes and assigned the participants to either group. The acupuncture therapists, rehabilitation therapists, and evaluator were unaware of this trial. All data were statistically analyzed by the first author, which effectively controlled the additional factors affecting the experimental results. There were no important changes to methods after trial commencement.

### 2.6 Intervention

The children in the conventional treatment group received behavioral and educational interventions, which was considered the primary treatment for ASD (18, 19), including applied behavior analysis (ABA) therapy, language training. and rehabilitation group training. ABA treatment is a one-to-one personalized treatment, with professional rehabilitation therapists providing behavioral intervention, cognitive therapy, social interaction, sensory and perceptual guidance, etc. according to the performance of children with ASD. Language training: one-to-one individualized training was adopted, which mainly included turn-based education, eye tracking, emotion regulation, picture exchange, motivation communication, play skills, Internet attention, mind interpretation, key response training, etc. Rehabilitation group training, including play therapy, music therapy, sensory integration training, behavior therapy, and sharing group, is jointly completed by rehabilitation therapists and parents. The above training was performed once a day, each treatment is 30 minutes, for 12 weeks, with 5 days of continuous treatment per week followed by 2 days of rest.

In addition to all the training of the conventional treatment group, the children in the acupuncture treatment group were also treated with scalp and tongue acupuncture. Sterile disposable acupuncture needles (0.3 × 4 cm; made in China-HWA-TO) were used for performing acupuncture. The scalp acupuncture points include Sishencong (EX-HN1), Baihui (GV20), Shenting (DU24), Yamen (DU15), Benshen (GB13), Yuzhen (BL9), Tianzhu (BL10), Speech Area 1, Speech Area 2, and Speech Area 3.Neiguan (PC6), Tongli (HT5), and Shenmen (HT7) are body acupuncture points, all located on the upper limbs (20).

The acupoints selected on the tongue were Naoming, Naoling, Bizhong, Naozhong. These acupoints are all extra points on the tongue. The EX-Tongue Naoming point is located bilaterally on the dorsum of the tongue, lateral to the central lingual midpoint. The EX-Tongue Naoling point is located at the midline of the tongue, 1 cun posterior to the tongue tip. The EX-Tongue Bizhong point is located at the midpoint of the median lingual fold on the dorsum of the tongue. The EX-Tongue Naozhong point is situated at the central midpoint on the dorsum of the tongue (21).

The children were instructed to sit still, and the needles were pushed with one or two hands at 15°–30°. They were pierced under the aponeurosis and parallel to the scalp 0.5–1 inch inside. A total of 20 acupuncture points are needled each time. Among these, scalp acupuncture points are retained for 1.5 hours.The needles were rotated twice every 30 min, known as “Xing Zhen” in Chinese. For body acupuncture points, as retaining needles might cause harm to children due to limb movement, a puncturing treatment method is used instead. Body acupuncture involves puncturing bilateral upper limb contralateral points. Tongue acupuncture also uses non-retention puncturing for stimulation points, which is not bloodletting therapy, and bleeding is minimized as much as possible. Acupuncture treatment for all ASD children was performed by the same acupuncturist with 20 years of acupuncture experience to locate the acupoints and perform acupuncture treatment. All children were treated at the hospital for 12 weeks, once a day, five days a week. The frequency and number of treatments were the same as the number of treatments in the conventional treatment group.

Special acupuncture strategies and cares for children with autism were adopted in this study. Considering children’s poor cooperation, emotional irritability and motor restlessness, all acupuncture procedures were performed with gentle, fast and shallow needling manipulation. For body acupoints, short retention time or non-retention needling was applied to reduce discomfort. For tongue acupoints, rapid insertion and immediate withdrawal were used to avoid choking risk and improve safety. Before needling, behavioral guidance and parental assisted soothing were conducted to relieve children’s anxiety. No excessive stimulation or painful manipulation was performed to ensure treatment safety and compliance in pediatric autistic patients. Nevertheless, we will still evaluate the children’s condition during the acupuncture treatment process. For example, continuous bleeding, fainting, infection, and persistent crying. If similar situations occur, provide timely medical treatment. Simultaneously communicate with the guardian to decide whether to conduct this study. Our team continues to provide focused attention.

### 2.7 Outcome measures

All children with ASD were assessed based on the CARS and PEP-3 scores before and after 12 weeks of rehabilitation treatment. CARS is a 15-item behavioural rating scale developed to diagnose autism in combination with clinical judgment. Additionally, it assesses the severity of the disorder. Studies have shown that CARS is a reliable and a stable indicator of autism in any child over 2 years of age as well as in adolescents. Each item is scored from 1 (no disease) to 4 (severe disease). A CARS score of more than 30 can diagnose ASD, and a score above 30 indicates a more severe degree of ASD. A score of 30–36 indicated mild-to-moderate ASD, and a score of 37–60 indicated severe ASD (13, 22). According to CARS scores, the conventional treatment group and acupuncture treatment group were divided into subgroups, the mild-to-moderate acupuncture subgroup, the mild-to-moderate conventional subgroup, the severe acupuncture subgroup, and the severe conventional subgroup. The developmental level and treatment effects on all children with ASD were evaluated using the PEP-3 scale. The PEP-3 is an assessment tool targeting young children with ASD between 2–7 years old across multiple skills. It can systematically analysis the characteristics of ASD children based on the data collected by the PEP-3 scale and relevant statistical methods (23). The former consists of 10 subtests and a total of 172 items to measure communication ability, motor ability, and maladaptive behaviors (24). The subsets include cognitive verbal/preverbal, expressive language, receptive language, gross motor, fine motor, and visual-motor imitation, affective expression, social reciprocity, characteristic motor behaviors, and characteristic verbal behaviors. Additional three subtests are in the Caregivers Report, problem behavior, personal self-care, and adaptive behavior. It has a three-prong scale system, with 0 = Fail, 1 = Emerge, and 2 = Pass. A higher score of PEP-3 indicates a better level of competence in children with ASD. In this study, the CARS and PEP-3 scores were used to evaluate the condition of children with ASD and the improvements in their functional progress. Although several studies have shown that acupuncture is safe in the treatment of ASD (9–11), we will still pay attention to the side effects of acupuncture in this study, such as local bleeding, infection, and persistent pain.

### 2.8 Statistical analysis

All data were analyzed using the Statistical Package for the Social Sciences (SPSS) 26.0 software (SPSS; Chicago, IL, USA). The categorical variables were described as frequency (n) and percentage (%) and were compared by performing the Chi-squared test. The continuous variables were described as the median and interquartile range (IQR), and the data were compared by performing the Mann-Whitney U test, as the data followed a non-normal distribution. All differences between the groups were considered to be statistically significant at *P* < 0.05.

## 3 Results

**Figure 1:**
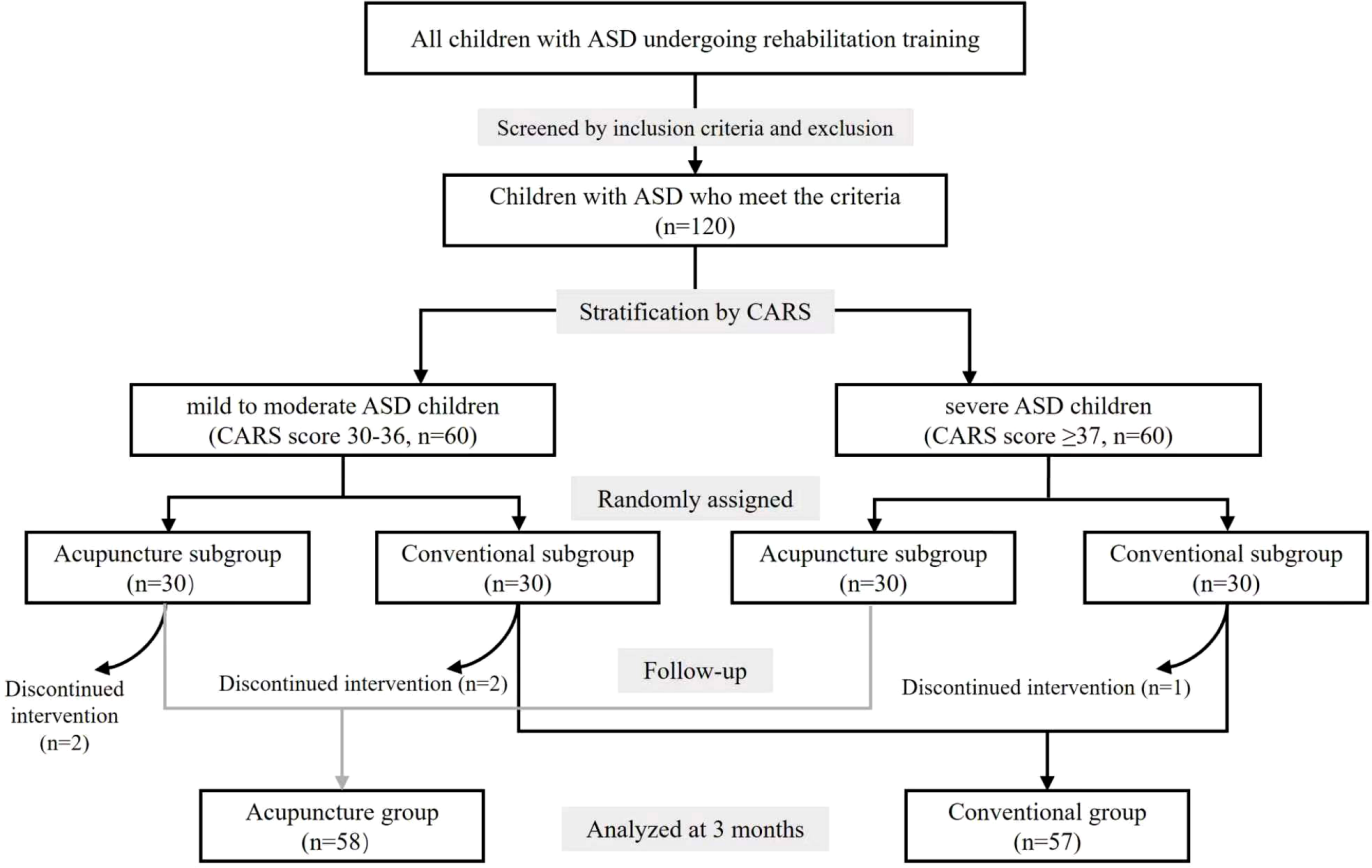
Flowchart for the participant selection process.

There were no changes to trial outcomes after the trial commenced. The flowchart for the process of selecting participants is shown in **Figure 1**. In total, 120 children with ASD participated in the experiment. Five children quit the study because of personal reasons or symptoms of other diseases during treatment. As for the 5 children with ASD who withdrew from this study, 2 of them were because their parents did not want to continue acupuncture treatment due to pain and crying. The other 3 children were hospitalized due to pulmonary infection during treatment, terminated rehabilitation training due to family reasons, and moved to other places too far away to continue treatment in our center. None of them stopped treatment because of adverse effects of acupuncture treatment. Finally, 115 children completed the experiments: 28 in the mild-to-moderate acupuncture subgroup, 28 in the mild-to-moderate conventional subgroup, 30 in the severe acupuncture subgroup, and 29 in the severe conventional subgroup. The baseline characteristics of the children in both groups are shown in **Table 1**. No significant differences were detected in the age and gender of the children between the acupuncture and conventional treatment groups (*P* > 0.05). The difference in the CARS and PEP-3 scores between the children in the conventional treatment group and the acupuncture group before the intervention was not statistically significant (**Table 1**).

**Table 1:** The baseline characteristics of the participants.

Characteristic	Conventional group (n=57)	Acupuncture group (n=58)	P-value
Gender, n(%)			0.265
Male	47(82.5)	52(89.7)	
Female	10(17.5)	6(10.3)	
Age, median[IQR]	34[30,41]	35[30,39]	0.525
Severity of ASD			
CARS, median [IQR]	37[34,41]	37[33,41]	0.761
CARS category, n (%)			0.928
Mild-to-moderate ASD (CARS < 37)	28(49.1)	28(48.3)	
Severe ASD (CARS ≥ 37)	29(50.2)	30(51.7)	
PEP-3, median [IQR]			
Cognition	12[7,22]	10[6,20]	0.166
Language expression	3[1,7]	2[1,4]	0.097
Language comprehension	6[1,15]	3[1,9]	0.169
Fine movement	21[15,26]	20[16,23]	0.600
Gross movement	16[12,21]	16[12,20]	0.993
Imitate	7[4,11]	6[4,10]	0.427
Emotional expression	7[5,11]	7[4,10]	0.447
Social interaction	5[2,7]	4[2,7]	0.846
Nonverbal behavior characteristics	15[7,17]	12[8,18]	0.931
Language behavior characteristics	2[0,6]	1[0,5]	0.742
Problem behavior	7[4,10]	4.5[3,8]	0.150
Self-care	11[9,15]	12[9,15]	0.405
Adaptive behavior	13[10,17]	13[11,16]	0.849

### 3.1 Overall results

**Figure 2:**
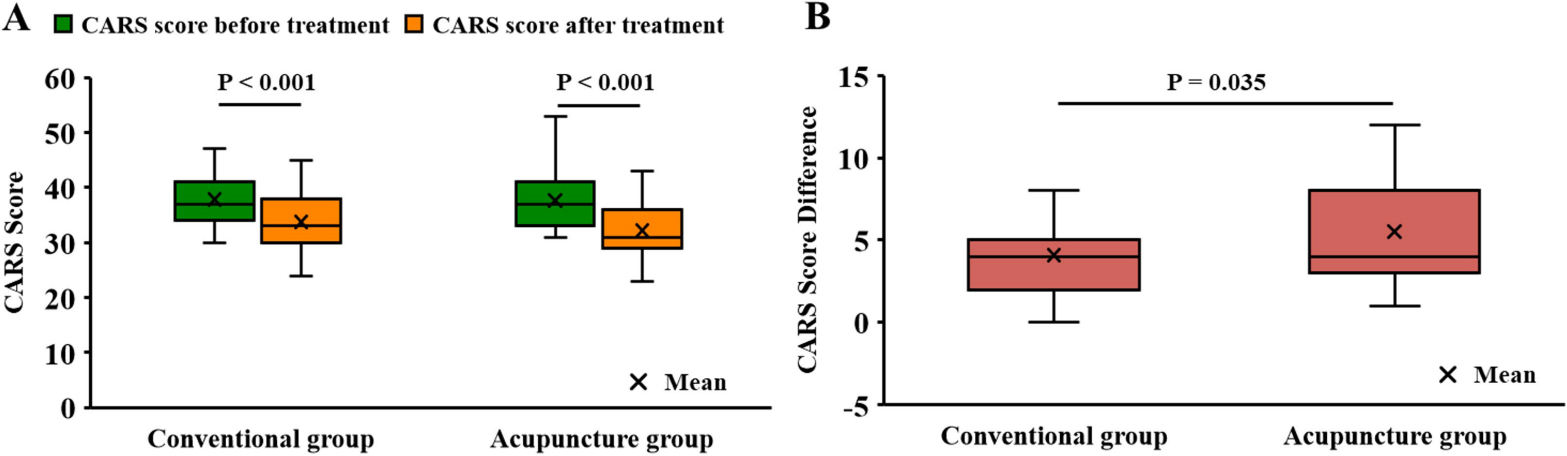
The observed values and changes from the baseline values for the CARS scores. **(A)** Difference in CARS values before and after treatment. **(B)** Comparison between two groups (P < 0.01; P>0.05, not significant).

After treatment, according to PEP-3 there were significant changes in the children of the conventional and acupuncture treatment groups compared to that before treatment, which showed that rehabilitation training, i.e., acupuncture and conventional treatment, affected the rehabilitation of children with ASD (**Table 2**). The same results from the CARS score can also be found (**Figure 2A**).

**Table 2:** The PEP-3 values before and after treatment of children in the conventional and acupuncture groups [median (IQR)].

Development Capability Dimension	Conventional group			Acupuncture group		
	Before treatment	After treatment	P-value	Before treatment	After treatment	P-value
Cognition	12[7,22]	24[18,33]	<0.001	10[6,20]	30[25,40]	<0.001
Language expression	3[1,7]	6[3,14]	<0.001	2[1,3]	10[5,16]	<0.001
Language comprehension	6[1,15]	15[10,26]	<0.001	3[1,9]	23[17,28]	<0.001
Fine movement	21[15,26]	28[24,33]	<0.001	20[16,23]	30[27,32]	<0.001
Gross movement	16[12,21]	25[20,28]	<0.001	16[12,20]	27[24,28]	<0.001
Imitate	7[4,11]	12[9,15]	<0.001	6[4,10]	14[11,16]	<0.001
Emotional expression	7[5,11]	11[10,14]	<0.001	7[4,10]	13[11,16]	<0.001
Social interaction	5[2,7]	10[8,13]	<0.001	4[2,7]	11[9,15]	<0.001
Nonverbal behavior characteristics	15[7,17]	20[15,24]	<0.001	12[8,18]	22[18,26]	<0.001
Language behavior characteristics	2[0,6]	8[5,10]	<0.001	1[0,5]	10[5,11]	<0.001
Problem behavior	7[4,10]	8[6,13]	<0.001	5[3,8]	10[6,12]	<0.001
Self-care	11[9,15]	14[12,17]	<0.001	12[9,15]	16[13,19]	<0.001
Adaptive behavior	13[10,17]	17[13,20]	<0.001	13[11,16]	18[15,23]	<0.001

We found that cognition verbal/preverbal, expression language, receptive language, fine motor, visual-motor imitation, social reciprocity, characteristic motor behaviors, problem behavior, and personal self-care of the children in the acupuncture treatment group were better than those of the children in the control group after treatment (**Table 3**). It could also be found from the CARS score that the treatment effect of the acupuncture group is more obvious than that of the conventional treatment group (**Figure 2B**).

**Table 3:** The changes in the PEP-3 values from baseline values for the children in the conventional and acupuncture groups [median (IQR)].

Development Capability Dimension	Conventional group	Acupuncture group	P-value
Cognition	12[2,19]	18[13,26]	<0.001
Language expression	2[1,8]	8[4,13]	<0.001
Language comprehension	7[3,15]	16[11,22]	<0.001
Fine movement	7[3,11]	10[7,12]	0.004
Gross movement	7[5,10]	9[6,12]	0.189
Imitate	4[2,7]	6[4,9]	0.003
Emotional expression	4[2,7]	5[3,9]	0.053
Social interaction	5[4,7]	7[5,9]	0.012
Nonverbal behavior characteristics	5[3,9]	8[4,12]	0.024
Language behavior characteristics	3[2,6]	6[1,9]	0.099
Problem behavior	2[0,4]	3[1,7]	0.027
Self-care	2[1,4]	3[2,5]	0.031
Adaptive behavior	2[1,7]	5[2,8]	0.076

### 3.2 Subgroup results

Similar results were found for the children in the mild-to-moderate group and the severe group. Rehabilitation training also strongly influenced children with different degrees of ASD (**Tables 4**, **5**).

**Table 4:** The PEP-3 values before and after treatment of the children with mild-to-moderate ASD in the conventional and acupuncture groups [median (IQR)].

Development Capability Dimension	Conventional group			Acupuncture group		
	Before treatment	After treatment	P-value	Before treatment	After treatment	P-value
Cognition	21[15,28]	28[17,41]	<0.001	20[10,24]	38[29,44]	<0.001
Language expression	6[4,10]	12[6,22]	<0.001	3[2,7]	15[7,26]	<0.001
Language comprehension	12[8,20]	24[12,30]	<0.001	6[3,18]	26[21,31]	<0.001
Fine movement	25[21,29]	31[27,34]	<0.001	24[20,28]	32[29,35]	<0.001
Gross movement	20[16,25]	27[24,28]	<0.001	20[16,23]	27[25,28]	<0.001
Imitate	11[7,13]	15[12,16]	<0.001	10[6,12]	16[12,17]	<0.001
Emotional expression	10[9,13]	13[10,15]	0.007	9[7,13]	14[11,17]	<0.001
Social interaction	7[5,10]	11[10,14]	<0.001	6[4,9]	13[10,17]	<0.001
Nonverbal behavior characteristics	17[15,21]	24[20,26]	<0.001	18[13,20]	24[20,28]	<0.001
Language behavior characteristics	5[2,10]	9[7,13]	<0.001	5[1,7]	11[7,12]	<0.001
Problem behavior	9[7,11]	12[9,13]	<0.001	8[4,9]	11[9,14]	0.001
Self-care	14[10,17]	16[13,19]	<0.001	14[10,17]	18[15,20]	0.002
Adaptive behavior	17[15,20]	20[15,22]	0.002	16[12,18]	21[17,24]	0.002

**Table 5:** The PEP-3 values before and after treatment of the children with severe ASD in the conventional and acupuncture groups [median (IQR)].

Development Capability Dimension	Conventional group			Acupuncture group		
	Before treatment	After treatment	P-value	Before treatment	After treatment	P-value
Cognition	7[5,10]	23[18,27]	<0.001	7[5,9]	26[23,31]	<0.001
Language expression	1[0,2]	3[2,7]	<0.001	1[0,2]	7[4,13]	<0.001
Language comprehension	1[0,3]	12[9,21]	<0.001	1[0,2]	20[14,24]	<0.001
Fine movement	17[13,21]	25[24,29]	<0.001	17[14,20]	29[26,30]	<0.001
Gross movement	12[10,16]	23[19,26]	<0.001	13[12,16]	25[21,27]	<0.001
Imitate	4[3,7]	10[6,12]	<0.001	4[3,5]	11[8,15]	<0.001
Emotional expression	5[3,7]	11[10,13]	<0.001	4[3,7]	13[9,14]	<0.001
Social interaction	2[1,4]	9[6,10]	<0.001	3[2,5]	11[7,12]	<0.001
Nonverbal behavior characteristics	7[5,13]	16[13,21]	<0.001	9[7,12]	20[16,23]	<0.001
Language behavior characteristics	0[0,3]	6[2,9]	<0.001	1[0,2]	7[3,10]	<0.001
Problem behavior	4[2,6]	7[4,8]	<0.001	3[2,5]	8[6,10]	<0.001
Self-care	9[7,13]	12[10,15]	<0.001	11[8,14]	15[12,17]	<0.001
Adaptive behavior	10[7,12]	15[11,17]	0.001	12[7,15]	17[14,20]	<0.001

#### 3.2.1 Mild-to-moderate ASD group

In the mild-to-moderate ASD group, acupuncture treatment significantly improved cognition verbal/preverbal, expression language, receptive language, visual-motor imitation, social reciprocity, and adaptive behavior (**Table 6**).

**Table 6:** The changes in the PEP-3 values from baseline values for the children with mild-to-moderate ASD in the conventional and acupuncture groups [median (IQR).

Development Capability Dimension	Conventional group	Acupuncture group	P-value
Cognition	4[1,14]	15[11,23]	<0.001
Language expression	3[0,11]	11[6,14]	0.012
Language comprehension	3[2,11]	16[11,22]	<0.001
Fine movement	6[3,8]	8[5,11]	0.057
Gross movement	6[5,7]	6[4,9]	0.760
Imitate	2[2,5]	6[4,8]	0.009
Emotional expression	3[2,5]	4[0,7]	0.157
Social interaction	5[3,6]	7[5,10]	0.006
Nonverbal behavior characteristics	5[4,7]	8[4,11]	0.085
Language behavior characteristics	3[2,5]	6[0,9]	0.273
Problem behavior	2[-1,4]	2[0,6]	0.373
Self-care	2[1,2]	4[1,6]	0.054
Adaptive behavior	2[1,3]	5[1,8]	0.037

#### 3.2.2 Severe ASD group

In the severe ASD group, acupuncture treatment significantly improved cognition verbal/preverbal, expression language, receptive language, and problem behavior (**Table 7**).

**Table 7:** The changes in the PEP-3 values from baseline values for the children with severe ASD in the conventional and acupuncture groups [median (IQR)].

Development Capability Dimension	Conventional group	Acupuncture group	P-value
Cognition	16[11,19]	20[14,26]	0.004
Language expression	2[2,6]	6[3,12]	0.002
Language comprehension	9[5,16]	17[11,23]	0.007
Fine movement	9[3,13]	11[9,15]	0.065
Gross movement	10[4,15]	10[8,13]	0.558
Imitate	5[2,8]	6[3,10]	0.132
Emotional expression	5[2,9]	7[4,10]	0.204
Social interaction	6[4,8]	7[4,8]	0.502
Nonverbal behavior characteristics	5[2,13]	9[5,13]	0.174
Language behavior characteristics	4[1,6]	6[2,9]	0.266
Problem behavior	2[0,4]	5[2,7]	0.028
Self-care	2[1,5]	3[2,5]	0.298
Adaptive behavior	4[0,9]	6[2,8]	0.632

### 3.3 Adverse reactions

No obvious adverse reactions were found in the whole process of acupuncture treatment, such as syncope, continuous bleeding, local infection, and continuous crying affecting other rehabilitation programs. In the process of acupuncture, some children with ASD will cry, but it can be sobered quickly and does not affect the emotional state in the future.

## 4 Discussion

In this study, we evaluated the effects of acupuncture and conventional rehabilitation treatment on children with different degrees of ASD. Acupuncture treatment has the potential to improve symptoms in children with ASD. Regardless of whether acupuncture treatment is used, rehabilitation training measures have a significant effect on improving the ability of children with ASD. These results are reflected in both PEP-3 and CARS scores. Moreover, the reduction in CARS scores was more pronounced in the acupuncture group than in the usual treatment group. The improvements in children with ASD can be further quantified using the PEP-3 scores. The combined administration of acupuncture and conventional rehabilitation therapy was more effective than conventional rehabilitation. Overall, acupuncture improved cognition verbal/preverbal, expression language, receptive language, fine motor, visual-motor imitation, social reciprocity, characteristic motor behaviors, problem behavior, and personal self-care in children of ASD. The therapeutic effects of acupuncture on children with different degrees of ASD were also different. Acupuncture treatment significantly improved cognition verbal/preverbal, expression language, receptive language, visual-motor imitation, social reciprocity, and adaptive behavior in children with mild-to-moderate ASD. Acupuncture positively affected cognition verbal/preverbal, expression language, receptive language, and problem behavior in children with severe ASD. In terms of the improvement of social interaction, acupuncture has a more obvious effect on children with mild to moderate ASD, which is conducive to guiding future rehabilitation treatment. There are different options for the treatment of children with different degrees of ASD.

For all children with ASD, acupuncture had positive effects on cognition, language expression, and language comprehension. This was mainly because the acupoints selected were associated with improvements in cognition and language. The EX-HN1, GV20, and DU24 acupoints mainly increase cognitive function; DU15, Speech area 1, Speech area 2, and Speech area 3, and tongue acupoints help in improving language function. Other acupoints, such as the auxiliary acupoints, have a calming effect; they reduce abnormal behavior, excessive excitement, and other conditioning effects (20).

Acupuncture has a certain physiological and imaging basis in the treatment of diseases (25, 26). As a basic test, acupuncture has been shown to be effective in treating ASD in animal experiments. In the USA, acupuncture has been used for effectively treating various diseases, especially chronic diseases (27). According to traditional Chinese medicine (TCM), the pathogenesis of ASD is related to the disharmony of five viscera, six bowels, and meridians, mainly the dysregulation of the heart, liver, spleen, kidneys, and brain (28). The organ and meridian concept in TCM is the fundamental basis for improving the behavior, cognition, and communication skills of children with ASD. Several studies have investigated the effect of acupuncture on children with ASD, including scalp acupuncture and tongue acupuncture (29, 30). These studies found that scalp acupuncture can improve functional levels in children with ASD, including language comprehension (29, 30), social interaction (30, 31), personal self-care ability (30), and adaptive behavior (32). Some studies have also shown that tongue acupuncture can improve language comprehension and social interaction (12). The results of these studies were similar to those of our study, and many of the selected acupoints were also similar. Scalp acupuncture was also found to decrease the CARS score and alleviate the symptoms of ASD (9, 20), similar to the results of our study. In this study, we found that acupuncture decreased CARS scores among children with severe ASD, which indicated that acupuncture can help to improve the overall ability of children with ASD (32). In addition to improving the overall function of children with ASD, acupuncture also has a certain alleviating effect on gastrointestinal problems that are prone to occur in children with ASD (33). We also found that acupuncture improved cognition and the effects were different for children with different degrees of ASD. The acupuncture points that we selected were related to the symptoms of ASD (15). Hence, these acupoints helped in improving cognitive functions. Compared with children with mild and moderate ASD, children with severe ASD showed greater improvement and greater recovery space after acupuncture treatment. This was the first study that categorized patients with different degrees of ASD into different groups for scalp acupuncture.

In this study, the main acupuncture points for acupuncture treatment included the scalp acupuncture points, while the tongue acupuncture point was used as an auxiliary treatment point. Tongue acupuncture can improve language function (12). Neiguan (PC6) and Tongli (HT5) are body acupuncture points. They are auxiliary acupoints and conform to the meridian theory of TCM. Speech area 1, speech area 2, and speech area 3 are the main acupoints for treating language dysfunction in TCM, and they can help improve language function (20). They are also the main points where acupuncture improved language expression and language comprehension in this study, and no international unified standard numbering is available.

Among all participants, five children quit the study; the parents of two children were unwilling to let their children participate in acupuncture treatment, while the other three children fell seriously ill during treatment, abruptly halting the rehabilitation process. Compliance with acupuncture treatment was high. A big problem we had to overcome was that many children cried or resisted during acupuncture, which necessitated the immobilization of the children with the assistance of parents to complete the treatment. The diameter of the acupuncture needle was 0.3 mm, and the pain was not obvious. After acupuncture, the children tolerated the needle retention process till the treatment was completed; the side effects were negligible. Some children will have a small amount of local bleeding, giving local pressure can stop the bleeding by themselves, without causing psychological burden to children and parents No infections or changes in the mental state, diet, and sleep patterns of the children were found. Overall, no specific adverse reactions occurred.

In this study, we administered scalp and tongue acupuncture treatment to 115 children with ASD (2–6 years old) and determined the effectiveness of the therapy. Acupuncture is a safe and mature traditional Chinese medicine therapy with a long clinical application history in ASD intervention. Characterized by mild stimulation and minimal side effects, it is highly suitable for children with poor drug tolerance. The combined acupoints used in this study, including scalp, tongue, body acupoints, specifically target core autistic symptoms such as language deficits, cognitive impairment and emotional disorders. By regulating cerebral circulation and neural activity, acupuncture improves children’s speech function and emotional stability. Compared with routine behavioral treatments, it achieves holistic mind-brain regulation with favorable compliance, presenting unique clinical advantages in pediatric ASD rehabilitation.

However, this study had several limitations. First, the autism diagnostic observation schedule (ADOS) and autism diagnostic interview-revised (ADI-R) are currently used to diagnose ASD. Since there was no change in the use of diagnostic tools, we used clinical observations and CARS scores; however, the accuracy of diagnosis needs further improvement. Additionally, we administered the treatment only for three months, which was short. Second, the efficacy evaluation depends mainly on behavioral scale assessment, lacking objective neuroimaging and biological indicators to clarify the underlying therapeutic mechanism.

Third, this was a single-center study and not a multi-center comprehensive experiment; thus, the reliability of the results is low. Complete double blindness is difficult because patients and guardians do not have access to blinding, and sham acupuncture was not performed in the control group. Making the study not a fully double-blind RCT. The continuous effect after acupuncture treatment was not monitored. Also, the influence of the degree of parental participation and guidance on the effect of rehabilitation treatment was not considered and needs further investigation. Future multi-center, large-sample, long-term follow-up studies are needed to further validate the clinical efficacy and mechanism of acupuncture for ASD.

## 5 Conclusion

Acupuncture benefited the functional level of children with different degrees of ASD. It improved cognition verbal/preverbal, expression language, receptive language, fine motor, visual-motor imitation, social reciprocity, characteristic motor behaviors, problem behavior, and personal self-care. In children with mild-to-moderate ASD, acupuncture along with comprehensive rehabilitation training improved cognition verbal/preverbal, expression language, receptive language, visual-motor imitation, social reciprocity, and adaptive behavior. In children with severe ASD, the combined treatment using scalp and tongue acupuncture with comprehensive rehabilitation training improved cognition verbal/preverbal, expression language, receptive language, and problem behavior. Additionally, the children cooperated during acupuncture treatment and did not show any side effects. Overall, our results suggested that acupuncture can be used to improve the condition of patients with ASD.

## Data Availability

The original contributions presented in the study are included in the article/supplementary material. Further inquiries can be directed to the corresponding author.
